# Glyceryl Trinitrate: History, Mystery, and Alcohol Intolerance

**DOI:** 10.3390/molecules26216581

**Published:** 2021-10-30

**Authors:** Russell Pearson, Anthony Butler

**Affiliations:** 1 School of Pharmacy & Bioengineering, Keele University, Newcastle-under-Lyme ST5 5BG, Staffordshire, UK; 2 School of Psychology & Neuroscience, University of St Andrews, St Andrews KY16 9JP, UK; arb3@st-andrews.ac.uk

**Keywords:** glyceryl trinitrate, NO-donors, organic nitrates, nitric oxide, aldehyde dehydrogenase, nitrate tolerance

## Abstract

Glyceryl trinitrate (GTN) is one of the earliest known treatments for angina with a fascinating history that bridges three centuries. However, despite its central role in the nitric oxide (NO) story as a NO-donating compound, establishing the precise mechanism of how GTN exerts its medicinal benefit has proven to be far more difficult. This review brings together the explosive and vasodilatory nature of this three-carbon molecule while providing an update on the likely in vivo pathways through which GTN, and the rest of the organic nitrate family, release NO, nitrite, or a combination of both, while also trying to explain nitrate tolerance. Over the last 20 years the alcohol detoxification enzyme, aldehyde dehydrogenase (ALDH), has undoubtedly emerged as the front runner to explaining GTN’s bioactivation. This is best illustrated by reduced GTN efficacy in subjects carrying the single point mutation (Glu504Lys) in ALDH, which is also responsible for alcohol intolerance, as characterized by flushing. While these findings are significant for anyone following the GTN story, they appear particularly relevant for healthcare professionals, and especially so, if administering GTN to patients as an emergency treatment. In short, although the GTN puzzle has not been fully solved, clinical study data continue to cement the importance of ALDH, as uncovered in 2002, as a key GTN activator.

## 1. Introduction

That nitric oxide (NO) has many roles in animal and plant physiology had lain undiscovered for many thousands of years was partly because it is such a small molecule and one that has a very short lifetime in a biological milieu. The discovery that it is a significant intermediate in the vitally important process of vascular smooth muscle relaxation was announced in 1987 in two seminal papers [1,2]. The discovery of a biological role for NO was greeted with some surprise and a little incredulity but the evidence is compelling. This evidence involved a demonstration that an aqueous solution of NO behaved biologically the same, with regard to smooth muscle relaxation, as the endothelium-derived relaxing factor (EDRF) discovered by Furchgott and Zawadzki in 1980 [3]. Glyceryl trinitrate (GTN) was used as a benchmark muscle relaxant with which NO and the EDRF were compared. Murad had established that GTN is a relaxant for vascular smooth muscle ex vivo, but the mechanism had not been studied in detail [4]. However, once the role of NO as a relaxant had been established, the presence of so many nitro-groups in GTN suggested its action might involve the formation of NO. However, there are chemical difficulties with this suggestion, although it appears so obvious. The transformation of nitrogen in a nitro-group into nitrogen in NO involves a 3-electron reduction, something unlikely to occur in one step. It is more probable there are two steps, one 2-electon step and a second one that is a 1-electron process [5]. The general chemistry and history of GTN and how it might generate NO in vivo are topics covered in this review.

There are two important factors to be borne in mind in any study of GTN. Firstly, GTN is a high explosive and produces oxides of nitrogen when it is detonated, but this is no guide to the mechanism of NO release under physiological conditions. Secondly, GTN does not spontaneously generate NO in the way, for example, that some *S*-nitroso compounds do (Equation (1)) [6]. Instead, such compounds could be muscle relaxants in vivo because of an accelerated release of NO under physiological conditions.

(1) $$2\mathrm{RSNO}\overset{\Delta ,hv\;{\mathrm{or}\;\mathrm{Cu}}^{+}\;}{⇌}\mathrm{RSSR}+2\mathrm{NO}$$

## 2. Invention of Glyceryl Trinitrate

The first synthesis of GTN is credited to the Italian chemist Ascanio Sobrero (1812–1888), a native of Piedmont. After studying chemistry in France and Germany he returned, in 1843, to Italy to take up a post at the Technical School in Torino. In Paris he had witnessed the successful nitration of cotton by the use of concentrated nitric acid, but when he tried this procedure on glycerine (1,2,3-trihydroxypropane) the product was oxalic acid. Clearly nitric acid had acted solely as an oxidising agent. However, use of a mixture of concentrated nitric and sulphuric acids gave Sobrero a different product, an oil that reminded him of olive oil and he called it pyroglycerine. Subsequent work showed that it was 1,2,3-trinitroxypropane or glyceryl trinitrate (GTN). It is generally referred to as GTN, although nitroglycerine is also commonly used. Sobrero found its most conspicuous property was its explosive action and he delayed making an announcement of his discovery because of the hazards in manipulation of the material. He was badly injured in one experiment.

## 3. Dynamite

Some years after its discovery GTN was taken up by the Swedish explosives entrepreneur Alfred Nobel [7]. He incorporated it into a diatomaceous earth (kieselguhr) to produce dynamite that was much safer to handle than GTN itself, although Alfred’s brother was killed in an accident during the development of the explosive. Dynamite is used extensively in civil engineering and Nobel deeply regretted its use in warfare. Such was the commercial success of dynamite that it contributed substantially to Nobel’s fortune from which he established the Nobel prizes. Although Nobel always gave credit to Sobrero for the invention of GTN, the latter felt he deserved greater recognition.

## 4. Biological Action of GTN

As was common practice at that time, Sobrero tasted the product he had made and the result was a severe headache that lasted several hours. Other members of the laboratory tried the same experiment with the same result. The reason for this effect was not obvious at the time, but it was not seen as an observation of value to humankind. However, it was of interest to one group of doctors, those who practised homeopathic medicine. During the 19th century homeopathic medicine was popular, although its scientific basis was slight and it is now largely discredited. One essential principle of homeopathic medicine is *similia similibus curantur*, ‘like cures like’. If GTN causes headaches then it must also be a cure for them.

As a prelude to its use in homeopathic medicine, it was subjected to a process of *Prüfung* or ‘proving’ by prominent homeopathic believers in which its biological properties were examined in some detail so that, when it came to be used clinically in regular medicine, some of the relevant safety considerations had already been examined. This is one of the few beneficial crossovers from homeopathic to allopathic prescribing.

## 5. GTN as a Muscle Relaxant

Angina pectoris is a medical condition typical of old people, but especially in elderly gentlemen of unseemly lifestyle. The conspicuous symptoms are breathlessness and an intense chest pain when the victim is hurried or emotionally distressed. The physician and physiologist Lauder Brunton found that he could relieve the pain of angina by venesection, although it did not cure the condition. This suggests that the condition could be caused by increased arterial tension, so he sought an agent that would lower blood pressure rapidly and reversibly. For this he chose inhalation of the recently invented compound amyl nitrite. More correctly, it is *iso*amyl nitrite, but the distinction is rarely made. That amyl nitrite did just that was discovered by a number of scientists, including Guthrie, Richardson, and Gamgee [8,9]. The last of these was a colleague of Brunton in Edinburgh. Clinical trials showed that it did, indeed, relieve the symptoms of angina rather well.

Amyl nitrite is not an ideal drug although it was regularly and frequently prescribed for the relief of angina and other conditions. Firstly, it is very volatile, and you can think you have a phial of amyl nitrite for emergency use when you have not. Secondly, the effect wears off very quickly. So, the search began for an alternative that did not have these shortcomings. It should be noted that no-one, at this time, seemed surprised that amyl nitrite had the effect it did and it would take another 100 years before any explanation was offered.

Amyl nitrite is still with us but with a somewhat different role. It was sold in the UK and the USA under the street name of ‘poppers’. When inhaled it not only relieves the symptoms of angina but also enlarges arteries to the brain and makes the recipient light headed. This, among some of the young, was equated with happiness. You can still buy it on the Internet under the name ‘Liquid Gold’, a rather extravagant name for a nostrum giving only a transient thrill, but such is the folly of humankind.

## 6. The Coming of GTN

Since its invention by Sobrero, it had been known that GTN was biologically active and it was soon established that its effect was due to a lowering of arterial tension, but the effect was variable and nonreproducible. The side effect of a severe headache was a barrier to its use in clinical medicine. Murrell, then a doctor at Westminster Hospital in London, decided to investigate the situation systematically in order to clarify matters. He experimented on himself at first, but later used a primitive sphygmometer to compare the effect of amyl nitrite and GTN on a patient’s pulse. With GTN the onset of pulse increase is slow but the effect is more sustained. Additionally, people varied in their response to the same dose of GTN and this may account for some of the early confusion. Headaches were avoided by keeping the dosage to a minimum, although for some sufferers a slight headache was an inevitable consequence of the use of GTN. By 1892 GTN had become the treatment of choice for angina relief and was included in the British Pharmacopoeia as a stock remedy. It is still in use today, over 100 years later. A liquid is not the most convenient form for a drug, and it has been suggested that it could be incorporated into a chocolate which be eaten either in one mouthful or nibbled when relief is required [10]. This suggestion does not seem to have been taken up.

The continued use of GTN in the treatment of angina indicates that it is a successful drug. For a long period, the mechanism of action remained a mystery with little speculation about it. With the discovery of a role for NO in the cardiovascular system, it seemed obvious that NO must be part of the mechanism of action. This is almost certainly correct but the remainder of the mechanism, the conversion of a 20 atom molecule like GTN into a diatomic molecule, is not a simple one-step process. More recent research has shed some light on this matter.

## 7. The GTN Conundrum

**Figure 1:**
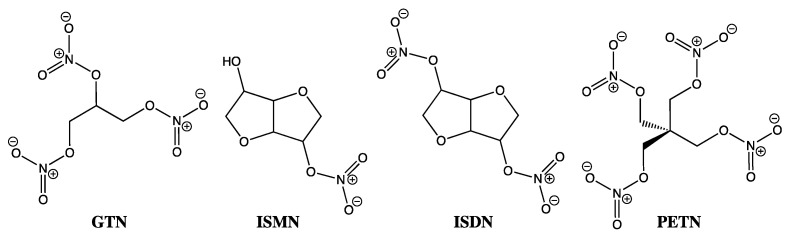
Chemical structures of GTN, ISMN, ISDM, and PETN.

Despite GTN usage by physicians since the late nineteenth century and still today in sublingual, transdermal, and slow intravenous infusions to routinely treat chest pain, heart failure, and heart attacks, its precise mechanism of action has remained something of a mystery. This conundrum extends beyond GTN and encompasses the entire organic nitrate family, including isosorbide mononitrate (ISMN), isosorbide dinitrate (ISDM), and pentaerythrityl tetranitrate (PETN) (Figure 1). As one of the most common classes of NO-donating compounds, organic nitrates, on first inspection at least, appear rather obvious in terms of their mode of action, with downstream events replicating those seen by other exogenous NO-donating species, where soluble guanylate cyclase (sGC) activation is followed by cyclic GMP-mediated relaxation. However, in similar fashion to the EDRF puzzle, which eluded us all over so many years before the Nobel Prize winning discovery of NO as the key biological messenger, by comparison, GTN has continued as a treatment and something of a tease over the past 150 years. Well beyond the 30th anniversary of the crucial NO discovery work [1,2,3,4,11,12], the crux of the GTN debate has rested on trying to understand its in vivo activity in terms of whether it primarily releases nitrite through a two-electron reduction, or nitric oxide by a three-electron reduction process, and in either case, how exactly is this achieved?

## 8. GTN Clearance versus Bioactivation

**Figure 2:**
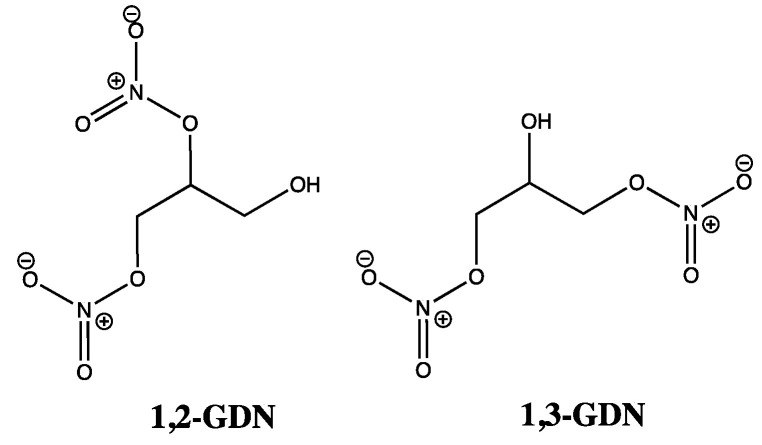
Chemical structures of 1,2-GDN and 1,3-GDN.

At this point, it is important to note that GTN and other organic nitrates do not activate sGC in cell-free systems [13], suggesting that any reduction processes predominantly occur by an enzymatic rather than a non-enzymatic mechanism. Thus, as prodrugs, GTN and other organic nitrates are said to rely on biotransformation mechanisms, which can be categorized under two distinct headings. The first deals with GTN clearance-based metabolism, with cell and organ bath studies suggesting 1,3-glyceryl dinitrate (1,3-GDN) (Figure 2) as the main product. On the other hand, chiral 1,2-glyceryl dinitrate (1,2-GDN) (Figure 2) is reported as the predominant product in GTN bioactivation or mechanism-based metabolism, with one GTN patch study reporting 1,2-GDN concentrations to be 2–7 times greater than for 1,3-GDN [5,13,14,15,16]. Such observations demonstrate regioselectivity rather than random denitration events, as GTN bioactivation could only favor 1,2-GDN over 1,3-GTN in a ratio of 2:1, if based purely on the available sites of attack, whereby two terminal nitrate groups at C1 and C3 of GTN would out-compete just one internal nitrate site (C2). In comparison, enzymatic degradation work has shown the opposite regioselectivity, including an anaerobic study confirming 1,3-GDN as the favored metabolite due to preferential attack at the secondary (C2) nitrate group [17]. Clearly the pathways linked to 1,2-GDN are of greatest interest here; however, for either type of biotransformation, what is less clear is whether NO, nitrite, nitrate, or a combination of all three accompany the formation of GDN.

## 9. Non-Enzymatic Reactions

For non-enzymatic GTN reactions, especially with thiols [18,19], the products are far clearer, with both 1,2 and 1,3-GDN generated [13], along with a stoichiometric amount of thionitrate (Equation (2)), which can then react with further thiolate or thiol, depending on the local pH, to generate nitrite (Equation (3)) or nitrous acid (Equation (4)), respectively [20,21,22,23]. In support of this pathway, the ISMN metabolite of ISDM has also been achieved in a model reaction with 2-mercaptoethanol at concentrations mimicking those seen by an enzyme [21]. The subsequent generation of dinitrogen trioxide by the slow bimolecular conversion of nitrous acid (Equation (5)) is questionable even under acidic conditions [24], but if this is achieved, free NO can be generated by homolytic cleavage of N_2_O_3_ (Equation (6)) or via nitrosation of thiol to yield *S*-nitrosothiols (Equation (7)), which can then homolytically cleave to release NO in the presence of heat, light, or copper, as seen previously by Equation (1) [25]. These reactions can therefore justify the formation of either nitrite or free NO. The reduction of nitrite to NO can also be achieved by ascorbate [26,27], but the mechanism involving RSNOs is far more appealing from the point of view of NO storage, to justify a more prolonged supply of NO in vivo. However, it has been reported that bioactivation of nitrite by non-enzymatic pathways, such as these, only becomes significant at millimolar concentrations of nitrite [13,28]. Reactions between cysteine and organic nitrates have also been shown to be highly dependent on temperature [29]. In addition, non-enzymatic reactions between GTN and either hemoglobin and myoglobin should also be considered as viable alternatives [30].

(2) $$\mathrm{GTN}+\mathrm{RSH}\underset{}{⇌}\mathrm{RSN}O_{2}+\mathrm{GDN}$$

(3) $$\mathrm{RSN}O_{2}+RS^{-}\underset{}{⇌}\mathrm{RSSR}+NO_{2}{}^{-}$$

(4) $$\mathrm{RSN}O_{2}+\mathrm{RSH}\underset{}{⇌}\mathrm{RSSR}+\mathrm{HN}O_{2}$$

(5) $$2\mathrm{HN}O_{2}\underset{}{⇌}N_{2}O_{3}+H_{2}O$$

(6) $$N_{2}O_{3}\underset{}{⇌}NO+NO_{2}$$

(7) $$N_{2}O_{3}+\mathrm{RSH}\underset{}{⇌}\mathrm{RSNO}+{\mathrm{HNO}}_{2}$$

## 10. Mechanistic Evidence from Observations Outside of the Lab

To ascertain how GTN and other organic nitrates function in vivo, there is a need to return to the very beginning of the GTN story and to focus on the individual testimonies and observations from as far back as the late 1860s. Within Alfred Nobel’s dynamite factories, workers complained of Monday morning headaches, while others suffering from angina or heart failure noted that chest pain was less severe on working days [31,32]. This relief from chest pain was explained by GTN’s vasodilatory action, while the Monday morning headaches uncovered the tolerance issues associated with GTN. The latter led to workers adding a small amount of GTN to their weekend hats to retain low levels of GTN exposure when not at work and in so doing, self-medicating to regulate a more consistent flow of blood to the head. However, headaches linked to GTN are still reported today, affecting 43% of patients when using 0.2% GTN ointments to reduce the symptoms of piles [33,34,35].

These two early observations, when taken alongside those from GTN-treated patients who complained of alcohol intolerance [21,22,36], represent three crucial and rather sizable non-lab based pieces in the GTN jigsaw puzzle, which can now be explained, at least in part, when placed together with the 2002 findings by Chen, Zhang, and Stamler [37]. This work built on studies by others in the 1970s and 1980s, who showed that GTN’s activity was the result of NO generated in vascular smooth muscle [20,38]. Chen and co-workers were able to expand on this by showing that mitochondrial aldehyde dehydrogenase (MtALDH or ALDH2) was the enzyme through which GTN was bioactivated [37], despite other enzymatic candidates also being suggested for this role, such as glutathione-*S*-transferase [39,40,41,42,43], cytochrome P450 [44,45,46,47,48], and xanthine oxidoreductase [49,50,51,52,53,54,55]. Central to the success of Chen and co-workers findings was the use of mouse RAW264.7 cells, grown in plentiful quantities, that allowed the identification of 1,2-GDN along with nitrite, rather than nitrate, to be confirmed from low, physiologically relevant quantities of GTN. Following on from the mouse model, the mitochondrial aldehyde dehydrogenase activity was further confirmed using a bovine liver model and also through comparative studies using reversible and irreversible ALDH inhibitors such as chloral hydrate and cyanamide, respectively [37].

## 11. Aldehyde Dehydrogenase (ALDH)

In hindsight, the alcohol intolerance experienced by GTN patients becomes easy to explain if GTN functions as both a substrate and an inhibitor for the same enzyme as that responsible for converting acetaldehyde to acetate, in the second step of ethanol’s metabolic pathway. In alcohol-related studies conducted between the mid-1980s and 1990s it was shown that GTN and ISDM, when taken by alcohol-consuming patients, led to nausea, blurred vision, and other symptoms synonymous with alcohol aversion therapies usually attributed to disulfiram, which is a known aldehyde dehydrogenase inhibitor [21,22,36,56]. 

**Figure 3:**
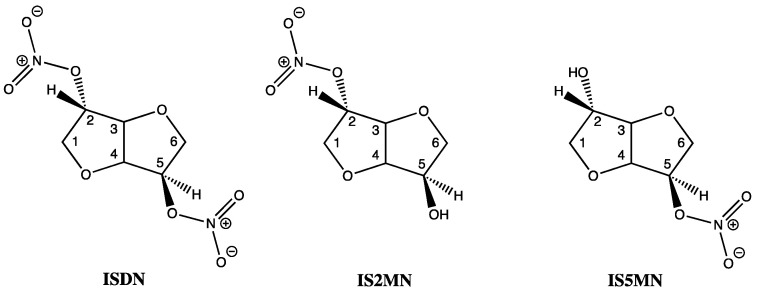
Chemical structures of ISDN, IS2MN and IS5MN.

Of the three known human aldehyde dehydrogenase isozymes, one is mitochondrial (E2 or ALDH2) and two are cytoplasmic (E1, or ALDH1 and E3, or ALDH3). In vitro studies, including saturation kinetics, involving E1 and E2 have shown that ISDM inactivated both isozymes by eradicating the enzymes dehydrogenase and esterase activities. ISDM along with its two hydrolysis products, isosorbide-2-mononitrate (IS2MN) as the minor and isosorbide-5-mononitrate (IS5MN) as the major product, as confirmed by HPLC, were all able to covalently inactivate both isozymes of aldehyde dehydrogenase at the active sites of E1 and E2 [21,22]. Interestingly, the bicyclic isosorbide core of ISDN forms a V-shaped wedge structure with both an exo- and endo nitrate ester, at the 2 and 5 positions, respectively (Figure 3). The lower inhibitory binding constants (Ki) for IS2MN and ISDN, compared to IS5MN, show that the nitrate at the 2-position is most important for recognition by the enzyme, and these values were also shown to be comparable to GTN when focusing on the E2 isozyme. However, rate constant data showed that GTN formed covalent bonds to E2 much faster than either the mono- or di-nitrates, indicating greater reactivity by GTN [21,22].

## 12. The Use of ALDH Inhibitors and ALDH Knockout Models

**Figure 4:**
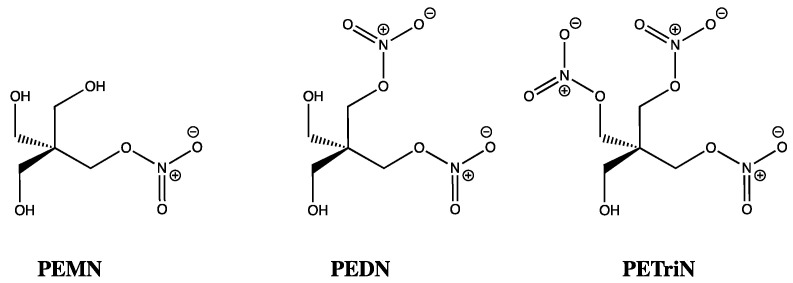
Chemical structures of PEMN, PEDN and PETriN.

While the inhibitory effects of ISMN, ISDN, and GTN on ALDH are fascinating, a discrepancy of sorts was uncovered once vasorelaxation was studied in mouse and rat models. Using aldehyde dehydrogenase inhibitors, such as daidzin and benomyl [57], bioactivation of organic nitrates with three or more nitrates, such as GTN, PETN, and pentaerythrityl trinitrate (PETriN), were shown to require ALDH2. In contrast, for those compounds with just one or two nitrates, such as ISDN, pentaerythrityl dinitrate (PEDN), and pentaerythrityl mononitrate (PEMN), their concentration-relaxation profiles were unchanged by ALDH inhibitors, suggesting bioactivation was independent of ALDH2 (Figure 4) [58]. This latter observation mirrors the findings of Chen and co-workers (2002), who did not see ISDN vasorelaxation diminished when using an ALDH2 knockout mouse model, whereas for GTN at low concentrations (up to 1 micromolar), ALDH2 played a central role [59]. Using GTN alongside the mono, di, tri, and tetranitrates of pentaerythrityl, Wenzel and co-workers (2007), using a similar ALDH2^-/-^ mouse model, showed that the number of nitrates rather than the carbon skeletal core was the dominant feature in determining which organic nitrates were reliant on ALDH2 for bioactivation [58]. However, as stated previously, using ALDH1, Pietruszko and co-workers (1995) did identify IS2MN and IS5MN as key metabolites from ISDN, by TLC and quantitatively by HPLC, which was reinforced by Tsou (2011) who showed NO release from ISDN due to ALDH1 [60]. This suggests either activity differences between isozymes or the existence of additional pathways for activation of ISDN that are ALDH independent [21,22]. Interestingly, ALDH3 has also been shown to denitrate GTN, ISDN, and IS2MN [61], while cytosolic ALDH2 has also been proposed to bioactivate GTN [62], as has ALDH1 [63].

## 13. The Active Site of Mitochondrial Aldehyde Dehydrogenase

In the active site of human ALDH2 (or mtALDH2), three important cysteine residues have been identified [37]. Cys-302 was first identified by Kitson and co-workers (1991) as an essential nucleophile for attacking aldehyde substrates in the esterase active site of cytoplasmic aldehyde dehydrogenase from sheep’s liver [64]. Interestingly, in 2009 Wenzl and co-workers reported that the ALDH2 mutant lacking Cys-302 showed over a 90% loss of enzyme activity towards GTN [65]. This is completely understandable since Cys302 is shown to be responsible for the nucleophilic attack preferentially at a terminal nitro group of GTN to generate thionitrate (RSNO_2_) and 1,2-GDN [37,66]. This accounts for the production of 1,2-GDN as the dominant metabolite, and, as such, the concentration of this dinitrate is often used as marker of GTN bioactivation, due to it being inherently more stable and reliably detectable in comparison to NO, nitrite, nitrate, or peroxynitrite. 

Cys301 and Cys303, which sit adjacent to Cys302, provide ideal sites for further nucleophilic attack, this time through an intramolecular process upon the newly formed thionitrate, and thus generating disulphide, which inactivates ALDH2 while releasing free nitrite. Evidence in support of disulphide formation at the active site comes from Shen and co-workers (2000), who were able to show, using electrospray ionization mass spectrometry, a 2 amu reduction in the enzyme’s molecular mass (Mr 4821 cf. 4823) when disulfiram was added to an in vitro model of rat liver mitochondrial ALDH [67]. A reduction in levels of free thiol groups from this homotetramer enzyme during its inactivation by organic nitrate was reported by Pietruszko and co-workers (1995) [22], albeit for ALDH1, who showed by titration with Ellman’s reagent an 80% drop in ALDH1 isozyme activity that was paralleled with the loss of two enzyme thiol groups.

**Scheme 1:**
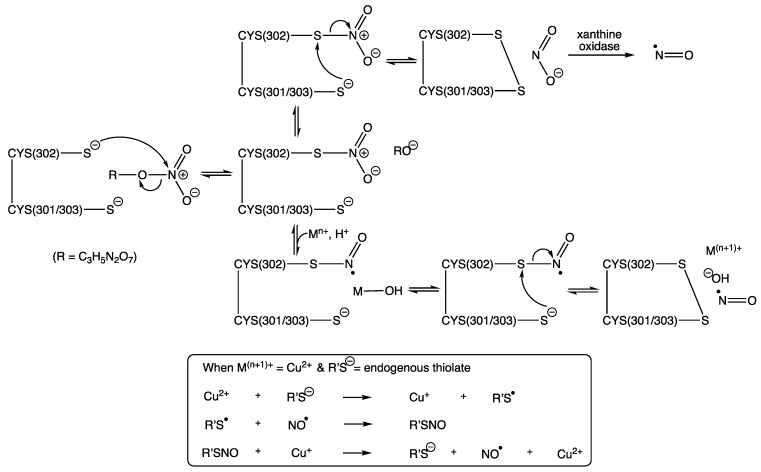
Two hypothetical pathways for GTN bioactivation (modified from work reported by Chen and Stamler (2006) [66] and from two of four pathways proposed by Mayer and Baretta (2008) [13]). R = C_3_H_5_N_2_O_6_.

Several hypothetical pathways for the denitration of GTN by ALDH2 have been proposed, resulting in either the generation of free NO or nitrite [5,13,14,15,20,37,66,68,69]. In 2012, Lang reported a crystal structure for ALDH2 with the thionitrate adduct seen on Cys302, which is stabilized by two hydrogen bonding interactions, the first with Asn-169 and the second with Cys302 itself, via the main chain amide [70]. This captures the product from first reaction step between ALDH2 and GTN, which can be seen in Scheme 1, alongside two proposals for the subsequent steps, both of which rely on the intramolecular attack by Cys301 or Cys303 to yield NO while creating the inactivated enzyme, due to the formation of the inert disulphide. Inactivation by a disulphide bridge via nitration of the active site was also supported by ^14^C-labeled ISDN studies (Pietruszko, 1995), which showed that the organic nitrates carbon skeleton was not bound within the enzyme [22]. In addition, and in support of previous findings, Lang was also able to use different conformations to support 1,2-GDN as the major metabolite formed in ALDH2′s active site and furthermore was able to propose the *R*-enantiomer of 1,2-GDN as the most likely chiral product formed [70].

Other pathways have suggested attack by hydroxide upon the thionitrate, from a water molecule activated by Glu268 [66]. However, this relies on an intermolecular reaction, which is likely to be out-competed by the intramolecular alternatives described in Scheme 1. Furthermore, Wenzl and co-workers (2009) created an ALDH2 mutant lacking Glu-268, which showed that whilst it is involved in the structural organization of the NAD-binding pocket it was not required for the bioactivation of GTN [65]. In addition, a further pathway describes the formation a sulphenyl nitrite RSONO, followed by O-N homolysis to give free NO, but this would result in sulphenyl radicals, which upon dimerization would surely lead to irreversibly inactivated ALDH2, rendering this also as an unlikely route in the bioactivation of GTN and other nitrates [13]. 

Support for the pathway involving metal complexation, as illustrated in Scheme 1, is especially attractive given the direct reduction to generate NO without the requirement for any further enzymatic steps. This, clearly, brings into question whether there would be a sufficient amount of transition metal to fulfil this role. However, the well-known significance of copper in the NO story, through its already proven ability to catalyze the release of NO from *S*-nitrosothiols, via Cu^+^ and not Cu^2+^, as established back in the 1990s (Equation (1)) [25], would surely make this a realistic candidate in this thionitrate chemistry, especially given its many mitochondrial roles through various copper-dependent proteins, including those linked to superoxide dismutase and cytochrome c oxidase [71]. 

The sustained activity of ALDH2 is clearly important for the bioactivation and metabolism of GTN and other organic nitrates. It has been shown that this activity can be restored by reductants, such as thiols able to reverse the disulfide bridge forming step between Cys302 and Cys301/303 (Scheme 1) [37]. Suitable reductants include dithiothreitol (DTT) [72,73], mercaptoethanol [22], cysteine, and *N*-acetylcysteine [12,31], but not glutathione [66,74]. In addition, ALDH2 can be partially restored by dihydrolipoic acid, which is a metabolic reductant present in mitochondria [13,74,75].

Returning to the findings by both Chen and Wenzel [58,59], it is feasible to expect both the number of nitrates on the parent compound, as well as the concentration of organic nitrate at the active site of ALDH, to be significant in terms of the amount of NO or nitrite produced. Data from Chen and separately by Wenzel, as already described, appear somewhat counterintuitive, since the lower nitrated organic nitrates (such as ISDN, PEMN, and PEDN) were least affected by ALDH inhibitors. The authors speculate that this somewhat confused picture could be the result of organic nitrates with three or more nitrates, having an increased likelihood of successfully nitrating one, two, or more cysteines in the active site of ALDH (such as Cys301, Cys302, and Cys303) thus forming multiple thionitrates. As a consequence, this may then alter the later mechanistic steps required for NO or nitrite release in the active site, which could potentially explain, at this point, the greater and more selective hindrance caused by ALDH inhibitors towards the higher nitrated compounds such as GTN, PETriN, and PETN.

## 14. Nitrate Tolerance

Vascular tolerance to GTN occurs if used continuously over 24–48 h [13]. It is for this reason that GTN patches are removed from patients overnight, with 10–12 h being the recommended time lapse before being reapplied [76]. While some reports suggest cross tolerance [66,77], where GTN treatment has been linked to the hindered activity of other classes of NO-donating compounds, this would seem unlikely as most other NO-donors release NO by very different mechanisms. However, others have explained this via GTN’s *S*-nitrosylation of sGC [78,79]. One problem when comparing GTN behavior with other NO donors is the lack of detectable free NO from GTN and also the lack of spectroscopic evidence from GTN to support the formation of the nitrosyl-haem complex, consistent with NO bound to sGC. The storage of NO in the form of *S*-nitrosothiols, as a NO congener, may help explain both of these anomalies, but for those who have instead directly measured NO levels created by GTN, this has led to some confusion. In 1996, Laursen measured NO levels created by GTN in both tolerant and control blood vessels and found comparable levels in each [80]. However, as reported by Mayer and Beretta (2008), instead of this signifying tolerance downstream of bioactivation, these results should instead be deemed inconclusive due to such low detectable levels of NO being characteristic across all such experiments involving GTN. In addition, while the downstream desensitization of sGC has been linked to GTN, this was only observed at GTN concentrations in excess of therapeutic levels and after prolonged exposure [13].

Other possible causes of nitrate tolerance include vascular thiol depletion [18,72], which is plausible based on the requirement of thiols to re-activate ALDH (Scheme 1), and when considering their central role in non-enzymatic thionitrate pathways (Equations (2)–(4)) and in the formation of RSNO (Equation (7)). In terms of optimum reactivity between cysteine and GTN to form nitrite and inert cystine and thus depleting thiol levels, this is known to occur most efficiently at pH 9.6, which closely mirrors the pH conditions required by aldehyde dehydrogenase [31]. For this non-enzymatic thiol chemistry in the mitochondria, this makes sense, since the thiol group of cysteine and glutathione have p*K*a values of 8.37 and 8.66, respectively, meaning both will predominantly exist in the ionized, reactive thiolate form under the pH environments required by mtALDH. Alternatively, such tolerance could be solely attributed to ALDH2 inactivation, due to disulphide bridging between Cys302-Cys301/303 in the enzyme’s active site, as already illustrated in Scheme 1.

The final consideration here acknowledges the generation of superoxide as a byproduct of GTN turnover by ALDH2, leading to a build-up of peroxynitrite, which forms the basis of the oxidative stress hypothesis to explain nitrate tolerance [81,82,83,84]. Validation for this as a cause of nitrate tolerance comes from the detection of superoxide, by hydroethidine [65], during GTN’s bioactivation by ALDH2. This is further supported by the use antioxidants such as vitamin E [85], ascorbate [86], and resveratrol [87], which were able to reverse tolerance, presumably through the depletion of superoxide, although such co-administration may also be responsible for both reversing the oxidative inactivation at ALDH2′s active site and protecting against endothelial dysfunction, as described by Mayer and Beretta [13]. A product of lipid peroxidation, 4-Hydroxynonenal, has also been linked with nitrate tolerance in more recent years [88].

## 15. GTN in Individuals with Lowered Alcohol Tolerance

Lowered alcohol tolerance due to the accumulation of acetaldehyde is seen in around 40% of the East Asian population. It is characterized by the subject flushing following alcohol consumption, due to the reduction of ALDH2′s dehydrogenase activity and is caused by a common mutation in the ALDH2 gene, involving a guanine to adenine polymorphism in exon 12 on chromosome 12. This single point mutation is therefore responsible for the replacement of glutamate, seen in the wild type, with lysine, as seen in the mutant, at amino acid position 487 (Glu487Lys) [89]. However, within the literature, the amino acid nomenclature can appear a little confusing here as the same mutant form of ALDH2 is also referred to as (Glu504Lys), due to the discovery of 17 additional amino acids on an *N*-terminus mitochondrial leader peptide, which were not previously included in the numbering system [90]. In short, these two descriptions relate to the same mutation and also explain why active site Cys302 is also reported as Cys319, which, as well as being the key nucleophilic base in the proposed bioactivation of GTN, is also the essential nucleophile for the dehydrogenase and esterase activity of the ALDH2 enzyme, which neatly explains the overlap between GTN bioactivation and alcohol metabolism [90].

In 1983, Goedde and co-workers conducted a study involving 1203 volunteers of different ethnicity, where the ALDH mutation responsible for alcohol sensitivity was investigated using skin and hair root samples [91]. Their findings showed clear ethnic variations. Highland Indians from Ecuador showed the greatest incidence of this ALDH polymorphism, which was identified in 69% of those studied, whereas in descending order, for Vietnamese, Japanese, Indonesians, Chinese, and volunteers from northern Thailand, the figures were 57%, 44%, 39%, 35%, and 8%, respectively. Incidences of this mutation dropped to zero in the remaining populations studied, which covered Europeans, Egyptians, Sudanese, Liberians, and Kenyans. Since this alcohol intolerance mirrors the effects of disulfiram, which is used as an adjunct to out-patient treatment of alcoholics, lower rates of alcoholism in populations with the Lys504 mutation can be clearly rationalized, as explained in this study [91]. 

While Glu504 homozygotes (ALDH2*1/1) have fully functional enzymatic activity, a drop to negligible levels is seen in Lys504 homozygotes (ALDH2*2/2) and only an uptick to 6% of normal activity is seen in Glu504/Lys504 heterozygotes (ALDH2*1/2) [92,93]. In light of GTN’s reliance on ALDH2 for bioactivation, as widely reported over the last 20 years, alongside evidence of higher plasma concentrations of GTN in subjects with the ALDH2 gene polymorphism [94], an obvious question to raise at this point is whether GTN therapy is somewhat compromised in subjects carrying the mutant gene. A number of studies have attempted to answer this with one of the more recent investigations conducted in Japan, in 2017, which concluded that Lys504 mutant subjects, when administered with GTN sublingually, showed no difference in terms of vasodilatory effects when compared to Glu504 wild type subjects [95]. This is reinforced by the findings of He and co-workers in 2019, following intra-arterial infusion of GTN [96]. In contrast, a Chinese study in 2015, also using sublingual GTN, involving 559 subjects, showed that cardiac output increased only in wild type subjects, regardless of whether they had coronary heart disease or not, but these values dropped in mutant subjects both with and without coronary heart disease, suggesting that GTN intervention may be significantly affected by the gene polymorphism [97]. In a separate Chinese study also using sublingual GTN, as reported by Li (2006) [90], for mutant subjects there was a lack of GTN efficacy, which was reinforced by enzyme kinetic studies that showed a 10-fold drop in GTN metabolism when using the Lys504 enzyme in place of the ALDH2 wild type. The latter finding maps nicely with the 10–20-fold drop in nucleophilicity at the active site Cys302/319 due to the presence of Lys504 [98].

Mackenzie and co-workers (2005) reported enhancements in forearm blood flow, using intra-arterial administration of GTN, but such improvements were still shown to be 40% lower in mutant subjects (*cf*. to wild type) [92]. This was mirrored by wild type responses to GTN following disulfiram therapy, which were attenuated by 33%, so in effect mimicking Lys504 mutant behavior, which led Mackenzie and co-workers to conclude that while important, under half of GTN’s total bioactivation was ALDH2 dependent. This aligns somewhat with Miura and co-workers’ (2017) description of alternative pathways for GTN bioactivation, as a way of explaining no vasodilatory differences in their own study [95]. In addition, at a purely molecular level, as a relatively small and flexible molecule with multiple polar bonds, GTN’s ability to access and either covalently or non-covalently bond in the active site of other enzymes certainly appears to be a very credible proposition.

Although alternative pathways do appear likely [99,100,101] and continue to be explored, the importance of ALDH2 in the bioactivation of organic nitrates cannot be overstated. Endorsement of this view is exemplified by Zhang and co-workers (2007) who recruited 142 coronary heart disease patients and showed that the amelioration of angina pectoris attacks within 5 min of GTN treatment was seen in 67.1% of patients who did not show flushing after drinking wine, compared to just 20.4% in flushing patients. This was consistent with genotype work, which showed that an efficacious response to GTN in 5 min was seen in 57.5% of Glu504 patients compared to 11.1% in the alcohol intolerant, Lys504 patients [102].

While reinforcing our understanding of GTN’s bioactivation, these clinical studies also show that, in addition to asking patients about their weekly alcohol consumption, healthcare professionals should also explore if their patients are taking any alcohol aversion therapy or experience any characteristic flushing following an alcoholic beverage [21]. Information on the latter could be viewed as a rather blunt diagnostic tool, but could actually be extremely useful in an emergency situation given that sublingual GTN is the standard treatment for acute angina pectoris [103], with others supporting the use of easily applied GTN patches to provide cardioprotection to at risk patients, during transportation in ambulances [104].

## 16. Conclusions

Although some of GTN’s bioactivation will be as a consequence of processes that are independent of aldehyde dehydrogenase, it is apparent that the active site of this alcohol de-toxifying enzyme plays a central role in governing the overall efficacy of GTN. It therefore follows that patients who are able to enjoy an alcoholic beverage without adverse alcoholic effects, when consumed in moderation, will also be amongst those most likely to experience the full benefits offered by GTN. That said, 174 years after its first synthesis in Turin, its complete fate once inside the body remains somewhat puzzling. In addition to other plausible enzymatic interventions occurring in tandem with aldehyde dehydrogenase, the significance of non-enzymatic in vivo reactions following administration of GTN should not be underestimated. In this regard, many of the remaining queries surrounding GTN and organic nitrates more generally, appear, at least in part, to resemble those uncovered whilst pondering in vivo reactions for *S*-nitrosothiols. As such, the biological cascades for these two separate classes of NO donor may yet be shown to converge in an even more complete manner than that already told, as the story of GTN enters the period beyond the 150th anniversary since both its explosive nature and clinical benefits were first realized.
